# Migration of Various Nanoparticles into Food Samples: A Review

**DOI:** 10.3390/foods10092114

**Published:** 2021-09-07

**Authors:** Saeed Paidari, Reza Tahergorabi, Ensieh Sadat Anari, Abdorezza Moahammdi Nafchi, Nafiseh Zamindar, Mohammad Goli

**Affiliations:** 1 Department of Food Science and Technology, Isfahan (Khorasgan) Branch, Islamic Azad University, Isfahan 81551-39998, Iran; s.paidari@srbiau.ac.ir (S.P.); Ensieh.Anari@gmail.com (E.S.A.); n.zamindar@khuisf.ac.ir (N.Z.); m.goli@khuisf.ac.ir (M.G.); 2 Food and Nutritional Sciences Program, North Carolina Agricultural and Technical State University, Greensboro, NC 27411, USA; 3 Food Biopolymer Research Group, Food Technology Division, School of Industrial Technology, Universiti Sains Malaysia, Minden 11800, Penang, Malaysia; amohammadi@usm.my; 4 Food Biopolymer Research Group, Food Science and Technology Department, Damghan Branch, Islamic Azad University, Damghan 36716-39998, Iran; 5 Laser and Biophotonics in Biotechnologies Research Center, Isfahan (Khorasgan) Branch, Islamic Azad University, Isfahan 81551-39998, Iran

**Keywords:** nanosilver, antimicrobial properties, clay, copper, silver, migration, food packaging

## Abstract

Nanotechnology has provided new opportunities for the food industry with its applications in food packaging. The addition of nanoparticles, such as clay, silver and copper, can improve the mechanical and antimicrobial properties of food packaging. However, nanoparticles may have an adverse impact on human health. This has led to legislative and regulatory concerns. The inhibitory effects of nano packaging on different microorganisms, such as Salmonella, *E. coli*, and molds, have been studied. Nanoparticles, like other materials, may have a diverse set of properties that need to be determined. In this review, different features of silver, clay and copper nanoparticles, such as their anti-microbial, cell toxicity, genetic toxicity, mechanical properties, and migration, are critically evaluated in the case of food packaging. Specifically, the viewpoints of WHO, FDA, and ESFA, concerning the nano-silver application in food packaging, are discussed as well.

## 1. Introduction

Food packaging aims to preserve the original quality of food products as a medium between products and the outside environment. It also provides consumers with information about the product. Preventing food decay and nutrient losses, preventing the spread of diseases caused by microorganism growth, and overall increasing the shelf-life of packaged foods, as well as preserving their quality, are among the most significant goals of food packaging [1,2,3]. Glass, metal, paper, foil, plastic, wood, and polymer laminate are some of the materials used for food packaging [4,5]. Polymer plastics are derived from petrochemicals, such as polyethylene, which is categorized as either high-density polyethylene (HDPE) or low-density polyethylene (LDPE); and polypropylene, polystyrene, and polyvinyl chloride are also used for food packaging [6,7,8]. Even though polymers have changed the food industry because of their high strength, low weight, low cost, water resistance, and easy processing, their most important limitations are their penetrability by oxygen and other gases and their non-biodegradability, which has led to the effort to find new technologies that overcome these limitations [9]. Nanotechnology might provide some solutions. It has been estimated that approximately 400 companies around the world have practical plans for using nanotechnology in foods and food packaging [10,11].

Active packaging systems are based on the interaction between the packaging environment and the food to provide proper protection. Active packaging using nanotechnology is being used to improve the quality and safety of food [12,13]. Such packaging can react to environmental conditions, such as temperature and moisture changes, and may in the future make consumers aware of the existence of contamination, pathogenic microorganisms and toxic materials. It also can sometimes recover after what normally would be destructive to regular packaging. Active packaging is also capable of releasing preservatives as soon as the food products start to decay [8,14]. Active packing is a new generation of dynamic food packing that slowly releases beneficial compounds or removes undesirable compounds [3,15].

Many antimicrobial materials can be used. To be successful, they often need to penetrate the food product at a level that is not harmful to consumers [16,17]. As particle sizes decreasd to the nanometer scale, their activity generally increases, particularly the speed with which they react with the surrounding environment, which usually improves their antimicrobial activity. Metals can be used with a variety of polymers such as either solid nanoparticles or metal oxides [18,19,20]. The most prevalent are nano-clay, nano-silver, and nano-copper.

Due to the high surface-to-volume ratio of nanoparticles, they have high reactivity to other materials; therefore, they have attracted much attention and have wide applications in the production of different goods. However, studies on nanoparticles have shown that some of them have negative effects on the growth and survival of creatures [21,22]. Nevertheless, there are various ambiguities about the toxicity mechanism of nanoparticles and, thus, recognizing these particles and their toxic effects is a necessity. In recent years, numerous studies have been conducted on the migration of nanoparticles to foodstuffs and, among them, due to governments’ concerns for the safety and health of silver nanoparticles, studies have been more concentrated on these nanoparticles. Ultimately, studies have demonstrated that nano-materials entering the body through different methods can be distributed throughout body organs and can damage human cells through changing mitochondrial function, producing active oxygen, increasing membrane permeability, and resulting in toxic effects and chronic diseases such as allergies, asthma, various inflammations, cardiovascular diseases and cancer [23]. Additionally, working with nanomaterials leads to the transmission of nanoparticles to the environment, which causes a kind of contamination called environmental pollution. No study has been performed on the mechanism of this transmission and its biodegradation. However, the presence of these nanoparticles affects the ecosystem [24]. The toxicity of nanoparticles is to a great extent linked to their physical and chemical properties such as size, shape, aspect ratio, density and chemical composition [25]. Studies have indicated that there is a reverse relationship between sizes of particles and their toxicity, as smaller particles have a high surface-to-volume ratio and thus their toxic effects are high due to the increase in their reactivity [26].

The most common material used in nanocomposites is modified clay soil. Modified clay soil is less expensive compared to other nano-materials as it is obtained naturally and is environmentally friendly [27,28]. The presence of clay nanoparticles in polymer chains generally strengthens the polymer network and improves the mechanical, thermal and inhibitory properties due to their strong interactions [29]. Copper is an essential mineral with strong antimicrobial effects on *E. coli*, Staphylococcus aureus, Enterobacter aerogenes and Propionic bacteria aeruginosa but it has less antimicrobial impact than silver [30]. Silver nanoparticles have the highest antimicrobial effect among metal nanoparticles to the extent that the strong microbicide affects a wide range of pathogens such as *E. coli*, molds, and viruses, but its usage is restricted due to the high cost of nano-silver particles. The important point is that the nanoparticles used in packaging might migrate into the food. Thus, their effect on food safety and human health must be considered [31,32,33,34].

## 2. General Rules for Using Nano-Materials in the Food Packaging

The laws and regulations of Europe and the United States substantially differ about nano-material packaging. European laws control all of the materials used in food packing that are in contact with food and have the potential to migrate into the food. All of the substances, which may either directly or indirectly touch the food, should necessarily be harmless. Therefore, there is a list of safe materials with safe doses. In the U.S., materials that do not pose any threat to consumer health and do not react with the food product, can be listed as safe substances. In other words, in American law, the dosage expected to be consumed by heavy use of relevant products determines whether they are considered toxic [35].

In the framework of European laws, the European Food Safety Authority (2021) determines the general principles for any materials that are in contact with food. However, there is no explicit mention of nanotechnology. European Union law (EU) No 10/20 deals with nano-materials that are mixed with food contact plastic materials [36]. As nanomaterials can have different physicochemical properties and, accordingly, different toxicological features to their normal counterparts that differ from larger structures, nanoparticles can only be used when explicitly allowed. Previous work with their larger counterparts does not cover nanoparticles. Moreover, producing a plastic layer with materials that are not made of materials listed in the European Union index is only allowed for non-food contact layers of the packaging. Risk evaluation of the nanoparticle packaging should be carried out on a case-by-case basis. As a result, nano-scale materials are categorized along with mutagens, carcinogens, and fertility toxicants [37,38].

The US FDA’s regulations about nano-materials published in 2014 [39] refer to particles between 1 nanometer and 1 micrometer. The policy is concerned with materials that are in contact with food. The Code of Federal Regulations (21 CFR) has no specifications for particle size, size distribution, or the morphology of indirect food additives (indirect) that are generally recognized as safe (GRAS). However, the FDA explained in an industry manual [39] that it does not consider nano-materials covered by these regulations. On the contrary, the FDA suggests that an important change in the production process with the use of nanotechnology might have an impact on the essence, safety, and supervision status of the material. Therefore, the FDA expects that safety evaluations should be based on the information obtained using the nanometer version [39,40].

Additionally, the FDA provided an industry guidance document entitled “The evaluation of whether the product adjusted according to FDA entails nano-technology or not”. The FDA intends to enforce the policies stated in the document. The FDA has declared that carbon, aluminum, nano-clay, and zinc oxide can be used as nanomaterials. However, to avoid legal liability, users generally require information from the manufacturer certifying that the product is made in compliance with the FDA guidance document.

In 2009, the Food and Agriculture Organization (FAO) and the World Health Organization (WHO) of the United Nations held a conference focusing on nanotechnology in the agricultural and food sectors. Experts from 13 countries participated and their consensus opinion and initial evaluations accepted the safety of nanoparticles [41].

## 3. Silver Nanoparticles

Silver atom particles from 1 to 100 nanometers are called silver nanoparticles [42,43]. These particles have different physicochemical properties and silver ions are better than other metal elements in many aspects [44,45]. They are not allergenic, have high stability, are environmentally friendly, and have antibacterial properties. Silver ions and combinations based on them have intrinsic antimicrobial, anti-mold, and anti-fungus properties against ~650 pathogens, which are killed in <6 min with contact with silver molecules, unlike many common antibiotics that can kill only five to six pathogens [46]. This is why silver nanoparticles are widely used in pharmaceuticals, plant, and animal agriculture products, cosmetics, and food sanitation [47]. The antimicrobial activities of silver nanoparticles are based on releasing silver ions formed using oxidative solutions [41]. Silver nanoparticles show their highest anti-microbial activity on *E. coli* and Staphylococcus aureus bacteria. They react significantly with substances in the cytoplasm as well as the nucleic acids in microbes to cause cellular disorders, destructions of their cell walls, and respiratory enzyme pathways. As a result, they either kill or inhibit the growth of microorganisms [48,49,50].

Polymer nano-composites produced with nano-silver have higher antibacterial activity and heat resistance; therefore, they are even more suitable for eliminating bacteria and fungus [51].

Ibrahim et al. (2021) investigated the antibacterial properties of silver nanoparticles in combination with cellulose base fabrics. According to the Disc diffusion method and colony count procedures, both Gram-positive and Gram-negative bacteria were reduced significantly [52]. Manikandan et al. (2021) assessed the antibacterial property of nano-composites containing green synthesized silver NPs on both Gram-negative and Gram-positive bacteria and the outcomes showed that its antimicrobial effect on *S. aureus* bacteria was greater than the effect on *E. coli* with it reacting in a dose-dependent manner in both cases [53].

## 4. Clay Nanoparticles

Montmorillonite (MMT) is a hydrated alumina-silicate layered clay consisting of an octahedral sheet of aluminum hydroxide between two silica tetrahedral layers [54]. Different types of nanoclay, such as MMT, Closite 15A, Closite 30B, and Closite 20A, can be used to produce nanocomposites. Moreover, various types of polymers, namely low-density polyethylene (LDPE), polyethylene, polyethylene terephthalate (PET), polylactic acid (PLA) and nylon, can be used to produce a nanocomposite. Recently, clay nanocomposites have been used along with chitosan, different plant extracts and essential oils in order to use their synergistic benefits in the packaging industry [18,55,56].

Clay nanoparticles are capable of reinforcing mechanical properties, for example, gas barrier characteristics and permeability to water, as well as the antimicrobial properties of biodegradable food packaging. Thus, many studies have been conducted in this arena and have attracted much attention from academia and industry. However, there is a concern with their safety in food applications. Therefore, migration studies are necessary to roll out this adverse impact [57,58,59].

Moreover, the reported antimicrobial properties of nanoparticles such as clay pose the question of whether they are hazardous to body cells, since legislative organizations, namely the FDA and EFSA, have set up rules about the migration of these nanoparticles. Food scientists have investigated different food materials and simulants to determine the potential hazards of different nanoparticles such as clay. Echegoyen et al. (2016) conducted a study on the evaluation of the potential migration of clay nanoparticles into ethanol 10% and acetic acid 3% as food simulants. Since clay is derived from different elements, Aluminum (Al) was chosen as representative of clay particles [35]. The results revealed that Al was released to the food simulants by a maximum of 51.65 ng/cm^2^ and 24.14 ng/cm^2^ for two different types of commercial clay packaging. However, the determination of the total percentage of used nanoclay is a key factor since it can change the migration rate significantly. Similar to Echegoyen, Farhoodi et al. (2013) measured the migration of Si and Al from clay packaging into acidic food simulants during 7 to 90 days of shelf life. The results showed that migration rates largely depended on storage time and temperature. In addition, Farhoodi et al. (2013) proved that the migration ratio of Si was 23% higher than that of Al. There are still some vague points about the migration of clay nanoparticles to food or food simulants since clay is derived from different elements, each of which can be released into food at different rates [60,61].

## 5. Copper (Cu) Nanoparticles

Copper was used as an antimicrobial agent many centuries ago. Ancient Egyptians and the Roman Empire are believed to be the first users of copper for the prevention of wound infections [62]. Since Copper ions are toxic to live cells, the application of copper nanoparticles has made them a proper agent for anti-microbial purposes. Numerous studies have shown that copper nanoparticles incorporated in nanocomposites could significantly inhibit pathogens [12,63,64].

Similar to silver and clay nanoparticles, copper also exerts its anti-microbial properties through the creation of radical oxygen, lipid peroxidation, and DNA degradation. The literature shows that copper particles have been incorporated into different matrixes, namely bovine serum albumin (BSA), Agar, carboxymethyl cellulose (CMC), cellulose, chitosan, low-density polyethylene (LDPE), high-density polyethylene (HDPE) and other matrixes [65,66,67,68]. A summary of different uses of nanoparticles in polymer matrixes is shown in Table 1. As a general rule, decreasing the size of nanoparticles increases their antimicrobial properties, although migration also increases. Increments of migration from one side are beneficial for exerting more antibacterial effects. However, it is hazardous to humankind as the legislation defines the maximum migration of 0.05 mg/kg for some nanoparticles.

Hannon et al. (2016) conducted a study on the migration of copper and silver nanoparticles into food simulants from the surface to measure potential migration using ICP–MS and prior acidic digestion methods. The results showed that 0.46 and 0.82 mg/kg of Ag and copper were released to the food simulants, respectively [65]. Cushen et al. (2014) studied the effect of time and temperature on the migration rates of Cu nanoparticles from the polyethylene matrix to chicken breast using ICP–MS. They showed that the ranges of migration were between 0.024 and 0.042 mg/dm^2^ [66].

## 6. Detection and Evaluation Methods of Nano-Materials

The detection and determination of the properties of nanomaterials in the food chain are mandatory due to the risks of particles for consumers as they have the potential to migrate from the packaging to food. Therefore, there exists a need for specific techniques to evaluate and analyze nano-materials [80]. To measure nanomaterials in complex matrices, analysis techniques should explicitly differentiate between nanoparticles and other matrix elements. Moreover, employed techniques should be sensitive enough so that they can detect low material concentrations and also supply sufficient information about the concentration, composition, and physicochemical properties of nanomaterials in samples. However, there is no chance to determine the real number of nanomaterials in food materials. In such complex chains, synthetic methods are required to determine the amount of migrated nanoparticles and detect them, and independent methods cannot supply all of the information [81]. The conventional chromatography methods are limited and inappropriate for polymer additives since they cannot measure the physicochemical properties of nanoparticles. Therefore, only a few methods are efficient for detecting nanoparticles and determining their properties. Different methods of nano-material detection are as follows:

### 6.1. Microscopic Methods

**Figure 1:**
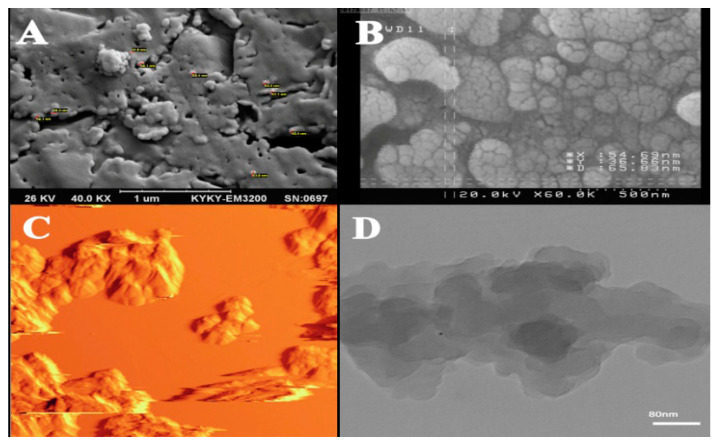
(**A**) SEM image of silver nanoparticle in LPDE polymer (Magnification X60), (**B**) FESEM image of coagulated copper nanoparticles (Magnification X60), (**C**) AFM image of silver nanoparticles (Scale of 80 nm), and (**D**) TEM image of silver nanoparticles.

High-resolution imaging methods, such as electronic microscopy (EM), are among the proper methods for detecting and depicting the shape, structure, size, and density of nanoparticles in the food matrix. Among such methods, transmission electron microscope (TEM), atomic force microscope (AFM), and scanning electron microscope (SEM) are more famous. These methods are capable of segregating nanoparticles down to one nanometer and are the only direct methods for studying the size distribution of nanoparticles [77]. Nevertheless, for obtaining comprehensive and sufficient information, more than hundreds of particles have to be counted, which is highly time-consuming, and as these methods have destructive effects on samples, samples under study cannot be evaluated using other methods [82]. Moreover, using the TEM method is suitable when the matrix under study is made of polymer. However, for detecting migration in complex environments such as in food or food-like stuff, other methods are needed [41]. As can be observed in Figure 1C, for obtaining 2-D information about nanoparticles in TEM, an electron is transmitted through particles. By comparing parts A and B in Figure 1, it is deduced that TEM has a lower resolution power than SEM.

### 6.2. Quantitative Analysis Methods

Methods for quantitative analysis include inductively coupled plasma-mass spectrometry (ICP–MS), atomic emission spectrometry (ICP–AES), and optical emission spectrometry (ICP–OES), which are among the most accurate and functional methods for determining metal nanoparticles. ICP–MS is superior to the other methods in this category due to its high sensitivity and potential for the detection and determination of metal quantity and selectivity [83]. Atomic absorption spectrometry is an alternative for ICP methods, which enjoys higher speed and sensitivity than ICP methods. However, the method is not successful for multi-elemental analysis. In a study by Song [83] on the migration of silver nanoparticles from polyethylene packaging to frozen food using ICP–MS, it was shown that the silver migration level in 3% acetic acid slightly increased over time and rising temperature; however, in 95% ethanol, the amount of nano-silver migration was dependent on time, and temperature changes showed no significant effects [83]. In another study, Lin et al. (2014) investigated nano-silver migration using ICP–MS and ICP–AES and realized that the most important stage in these tests was sample digestion. They also reported that ICP–MS enjoyed a higher precision in comparison with ICP–AES for detecting the migration of silver nanoparticles.

### 6.3. Spectroscopy Methods

Spectroscopy methods include X-ray Diffusion (XRD) and Ultraviolet-visible Spectroscopy (UV-VIS) that are used for obtaining information such as elemental composition or structure and accumulation of nanomaterials. These methods have vast applications due to being destructive. Spectroscopy methods, due to low costs and easy usage, are used as supportive techniques [84,85].

#### D-Titration and Migration

In research conducted by Hosseini et al. (2017), titration and migration methods demonstrated different percentages of silver nanoparticles remaining in packaging covers containing 5–8% nanoparticles. Although there was no significant difference between emitted nanoparticles with 1 or 3 percentages in packaging with 1–3% nanoparticles, there was a statistically significant difference between the two methods employed in this study in those containing 5% and 8% nanoparticles. Observations showed that the release amount of silver nanoparticles through titration was zero on days 0–6 in 1–3% treatments and that the highest release level was in the 8% treatment on day 6 with 6 ppm. Moreover, the release amount of nanoparticles through the migration test was reported to be zero on days 0–6 in 1–3–5% treatments containing silver nanoparticles, and that the highest release level was observed in the packaging containing 8% silver nanoparticles on days 5 and 6. Based on the results obtained in the study on the release amount of nanoparticles using the titration and migration methods, the titration method had a higher sensitivity in terms of measuring the released nanoparticles, compared to the migration method. The titration method manages to provide more precise information as compared with the migration method, and allows the determination of nanoparticles released from polymer packaging covers exposed to heat [67]. The migration method is believed to be an old method used for other packaging. However, based on the American National Standards Institute EN-1186 and 11737-3, this is the best-suggested method for nylon and polystyrene packaging.

## 7. Conclusions

Packaging containing nanoparticles can enhance the shelf-life of products and hence decrease waste and its negative effects on the environment. Although the packaging process can be a source of chemical contamination of foods, the amount of materials in packaging that migrates to the food surface is also an important factor in food packaging. Many studies have shown the migration of nanomaterials from packaging to the food matrix. However, some of these studies have experimentally demonstrated migration levels to be less than the permitted threshold. It has been observed that studies on the migration of silver nanoparticles are not congruent with each other; however, all studies in this regard agree upon the fact that silver nanoparticles have high migration levels in acidic environments. Currently, the physicochemical properties of nanoparticles, the methods for detecting the migration of nanoparticles and quantifying them in food, and the relationship between properties of nanoparticles and their toxic effects, are some of the most important subjects to be studied. Moreover, studies have shown that the migration rate of materials depends on numerous factors such as the density of remaining segments, the thickness of additives, the essence of the foodstuff in contact with nanoparticles, the solubility of the materials present in food, and the duration for, and heat in which packaging materials and food are in contact with each other. In general, nanoparticles are capable of easily migrating to cell units of foods. However, information is still insufficient about the toxicity of these materials, although it is being constantly updated.

## Figures and Tables

**Table 1:** A summary of migration studies on NPs.

NP	Matrix	Sample or Simulant	Detection Method	References
Silver	PP	Ethanol 10% & Acetic Acid (AA) 3%	Titration	[67]
Silver	LDPE	Sturgeon (Bulga)	Titration	[68]
Silver	LDPE-PT	Milk	ICP-AES	[65]
Silver	LDPE	Chicken Breast	ICP-Ms	[69]
Silver	LDPE	Ethanol 10%, AA 3%, Olive Oil	ICP-Ms	[70]
Silver	LDPE-PT	Ethanol 10%, AA 3%	ICP-Ms	[71]
Silver	LDPE	Ethanol 10%, AA 3%	ICP-Ms	[72]
Silver	LDPE-PP	Ethanol 10%, AA 3%	ICP-Ms	[73]
ZnO	LDPE	Chicken Breast	ICP-Ms	[69]
Clay	PP	Cheese water	ICP-Ms	[74]
Clay	LDPE	Ethanol 10%, AA 3%	ICP-Ms	[73]
Clay	PLA	AA 3%	ICP-OEs	[60]
Clay	PLA	Ethanol 95%	ICP-Ms & AF4	[75]
Ti	LDPE	Deionized water, Ethanol 10%, AA 3%	ICP-MS	[76]
Ti	LDPE	95% Ethanol	ICP-MS	[77]
Cu	LDPE	Ethanol 10%, AA 3%	ICP-MS	[78]
Cr	Stainless Steel	AA 3%	ICP-Ms	[79]
Fe	Stainless Steel	AA 3%	ICP-Ms	[68]

Silver, clay, titanium, copper, chromium, and iron particles were used in different studies while their size was <100 nm.

## Data Availability

Not applicable.
